# Maternal treatment with a selective delta-opioid receptor agonist during gestation has a sex-specific pro-cognitive action in offspring: mechanisms involved

**DOI:** 10.3389/fphar.2024.1357575

**Published:** 2024-04-16

**Authors:** Eliyahu Dremencov, Henrieta Oravcova, Daniil Grinchii, Zuzana Romanova, Roman Dekhtiarenko, Lubica Lacinova, Daniela Jezova

**Affiliations:** ^1^ Institute of Molecular Physiology and Genetics, Centre of Biosciences, Slovak Academy of Sciences, Bratislava, Slovakia; ^2^ Institute of Experimental Endocrinology, Biomedical Research Center, Slovak Academy of Sciences, Bratislava, Slovakia

**Keywords:** delta opioid receptor (DOR), prenatal treatment, dopamine, glutamate, hippocampus, electrophysiology, novel object recognition (NOR)

## Abstract

**Background:** There is growing evidence that the treatment of several mental disorders can potentially benefit from activation of delta-opioid receptors. In the future, delta-agonists with a safe pharmacological profile can be used for the treatment of mood disorders in pregnant women. However, the data on prenatal exposure to delta-opioid agonists are missing. The present study is aimed to test the hypothesis that the activation of delta-opioid receptors during gravidity has positive effects on the behaviour accompanied by changes in glutamate and monoamine neurotransmission.

**Methods:** Gestating Wistar rats were chronically treated with a selective delta-agonist SNC80 or vehicle. Adult male and female offspring underwent novel object recognition (for the assessment of cognition) and open field (for the assessment of anxiety and habituation) tests, followed by *in vivo* electrophysiological examination of the activity of hippocampal glutamate and midbrain serotonin (5-HT) and dopamine neurons.

**Results:** We found that the maternal treatment with SNC80 did not affect the offspring’s anxiety, habituation, and 5-HT neuronal firing activity. Female offspring of SNC80-treated dams exhibited improved novelty recognition associated with decreased firing rate and burst activity of glutamate and dopamine neurons.

**Conclusion:** Maternal treatment with delta-opioid agonists during gestation may have a pro-cognitive effect on offspring without any negative effects on anxiety and habituation. The putative pro-cognitive effect might be mediated via mechanism(s) involving the firing activity of hippocampal glutamate and mesolimbic dopamine neurons.

## 1 Introduction

For a long time, psychopharmacotherapy during pregnancy has been a contradictory issue. It is hardly possible to get full evidence to decide, whether the treatment with psychotropic drugs represents a lower risk than the mental disease itself. This is particularly important for patients with depressive and anxiety disorders. American College of Obstetricians and Gynaecologists recommends using selective serotonin reuptake inhibitors or serotonin-norepinephrine reuptake inhibitors in pregnancy, as first-line pharmacotherapy, due to their low rates of malformations (ACOG Practice Bulletin, 2008). Prescribed antidepressants cross the placenta, and they may increase the levels of monoamines in the brain (Nordeng et al., 2001; Laine et al., 2003). These can affect the functional development of the brain and the behaviour of the child. On the other hand, untreated depression can adversely affect maternal health and may lead to maternal-child relationship disturbances (Dubovicky et al., 2017; Idunkova et al., 2023). Therefore, an unmet need of pharmaco-psychiatry is the development of antidepressant and anxiolytic drugs with a safe profile.

There is growing evidence that the treatment of several mental disorders can potentially benefit from affecting opioid receptors. The endogenous opioid system may be dysregulated in depressive disorders and drugs targeting mu-, delta-, and kappa-opioid receptors have antidepressant potential. Of interest are the delta-opioid ligands because they have a different pharmacological profile than mu- and kappa-opioid agonists (Jelen et al., 2022). Indeed, preclinical studies demonstrate the ability of delta-opioid receptor agonists to induce positive effects on anxiety and depression-like behaviour (Saitoh et al., 2004; Jutkiewicz et al., 2005). We have shown in rats that chronic treatment with the delta-opioid agonist SNC80 induced mild anxiolytic effects and increased locomotion. The behavioural changes were accompanied by alterations in central neurotransmission as revealed by *in vivo* electrophysiology (Dremencov et al., 2023).

Moreover, the preclinical studies also suggest the ability of opioid receptor ligands to modulate cognitive processes (Jacobson et al., 2018). The mechanisms involved in the cognitive and other behavioural effects can be related to monoamines and other central neurotransmitters, neurogenesis, neuroplasticity, and neurotrophic factors (Jelen et al., 2023). It has been shown that delta-opioid agonists can increase the proliferation of hippocampal progenitor cells (Persson et al., 2003a), while the treatment with delta antagonists induced an opposite effect (Persson et al., 2003b). The positive effects of delta-opioid agonists on gene expression of brain-derived neurotrophic factor (BDNF), known to be important for processes of learning and memory, have also been described (Torregrossa et al., 2006).

Based on current preclinical and early clinical studies, new agonistic molecules acting at delta-opioid receptors are being developed with satisfactory antidepressant/anxiolytic effects without the risk of seizures and other significant adverse events (Jelen et al., 2022). In the future, such delta-agonists with a safe pharmacological profile can be used for the treatment of mood disorders in pregnant women. However, the data on prenatal exposure to delta-opioid agonists are missing. The present study is aimed to test the hypotheses that (i) the activation of delta-opioid receptors during gravidity has positive effects on the behaviour and (ii) behavioural changes are associated with alterations in glutamate and monoamine neurotransmission. We have focussed in glutamate neurons of the hippocampus, serotonin (5-HT) neurons of the dorsal raphe nucleus (DRN), and dopamine neurons of the ventral tegmental area (VTA) because of the key roles played by these neurons and brain areas in both cognitive and emotional behaviour (Lyon et al., 2012).

## 2 Methods

### 2.1 Drugs and chemicals

The SNC80 was purchased from Bio-Techne Ltd., Abingdon, United Kingdom, and other drugs and chemicals from Merck Life Science s. r.o, Bratislava, Slovakia. Chloral hydrate and urethane were dissolved in saline (0.9% sodium chloride). SNC80 was dissolved in 1M hydrochloric acid. The SNC80 solution was next diluted with saline (1:100) and adjusted to pH 7 with sodium hydroxide.

### 2.2 Animals

Nulliparous Wistar rats, weighting 200–220 g, were obtained from the Breeding Facility of the Centre of Experimental Medicine of the Slovak Academy of Sciences Dobra Voda, Slovakia). Animals were kept under standard conditions with a 12:12 h light/dark cycle (lights on at 7 a.m.), temperature (22°C ± 2°C), and humidity (55% ± 10%). All experimental procedures were approved by the Animal Health and Animal Welfare Division of the State Veterinary and Food Administration of the Slovak Republic (Permit number Ro 4103/18-221/3) and conformed to the Directive 2010/63/EU of the European Parliament.

### 2.3 Mating and SNC80 treatment

Female rats were mated with males in a ratio of 3:1. The presence of spermatozoa in vaginal smears was considered day zero of gestation. SNC80 (2.5 mg/kg/day) or vehicle (1M hydrochloric acid diluted with saline 1:100, pH ≈ 7) were injected subcutaneously, every day between days 11th and 19th of the gestation. There were 11 dams in each treatment group. The dose of SNC80 has been selected according to our previous dose-response study (Grinchii et al., 2023). Similarly, the period of gestation was chosen according to previous experiments with antidepressant drug treatments during gestation (Viñas-Noguera et al., 2022; Grinchii et al., 2024).

### 2.4 Offspring study design

**FIGURE 1:**
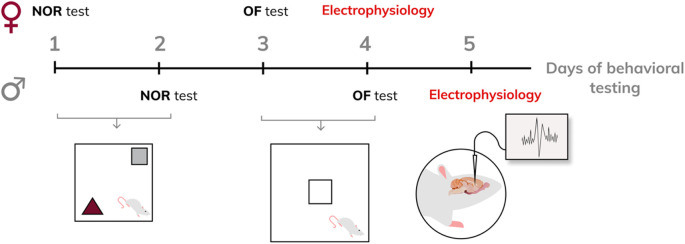
Experiment design.

The offspring were weaned on *postpartum* day 21 and housed in litter groups of the same sex (four animals per cage). Rats reaching adulthood first underwent the novel object recognition (NOR) test. Forty-8 hours after the NOR test, the same animals were subjected to the open field (OF) test. Twenty-4 hours after the OF test, the same rats were used for *in vivo* electrophysiological recordings. At the end of electrophysiological measurements, the animals were euthanized by an overdose of urethane or chloralhydrate. A scheme of behavioral testing is provided in Figure 1.

### 2.5 Novel object recognition (NOR) test

To investigate learning and memory, adult offspring were subjected to the NOR test, as previously described (Hlavacova et al., 2015). The black plastic apparatus (Ekoplast, Telc, Czech Republic) consisted of a 50 cm × 50 cm square-shaped arena with 40 cm high walls was used. The arena was illuminated by dim light with an intensity of 50 lx. The two identical objects were placed in the arena before the test. Each animal was gently put in the corner of the arena and allowed to explore the objects for 5 min. After that, the rat was placed in a separate cage for 3 min while the apparatus and objects were cleaned with 20% ethanol and water. One of the familiar objects was replaced by a new one and the animal was placed back in the arena for another 5 min. The last 5 min of the test were evaluated in the computer program H77, Budapest, Hungary. The time spent exploring each object was recorded. Based on these values, the ratio of the time of recognition of a new object to the sum of the time of recognition of a new and an old object was calculated (recognition index).

### 2.6 The open field (OF) tests

The anxiety behaviour, locomotor activity and habituation processes were evaluated in adult offspring. The OF apparatus (Ekoplast, Telc, Czech Republic) which consisted of a rubber square area of 100 × 100 cm surrounded by a plastic 52 cm high walls was used for the performance of the OF tests. The illumination intensity was 40–45 lx in the central zone and 15–25 lx in the peripheral area of the apparatus. To begin the test, each rat was placed in the corner of the OF. The OF test lasted 15 min. The movement of the rat was continuously recorded using a video camera. The records were analyzed using the H77 program (Institute of Experimental Medicine, Budapest, Hungary).

The number of entries and time spent in the central zone of the OF were evaluated as measures of anxiety behavior. Four paws entering the central zone were defined as a central zone entry.

To assess the total locomotion activity and habituation processes, the OF arena was divided into 36 squares grid. The number of crossed squares was recorded. Average values of squares crossed in the first, second, and third 5-min periods of the test were calculated according to the approach published in our previous studies. Linear regression was used to evaluate the habituation course of locomotor activity. The individual rate of habituation (*k*-value) expresses the rapidity of habituation (Dubovicky et al., 1999; Dubovicky and Jezova 2004; Dremencov et al., 2023).

### 2.7 Electrophysiology *in vivo*

**FIGURE 2:**
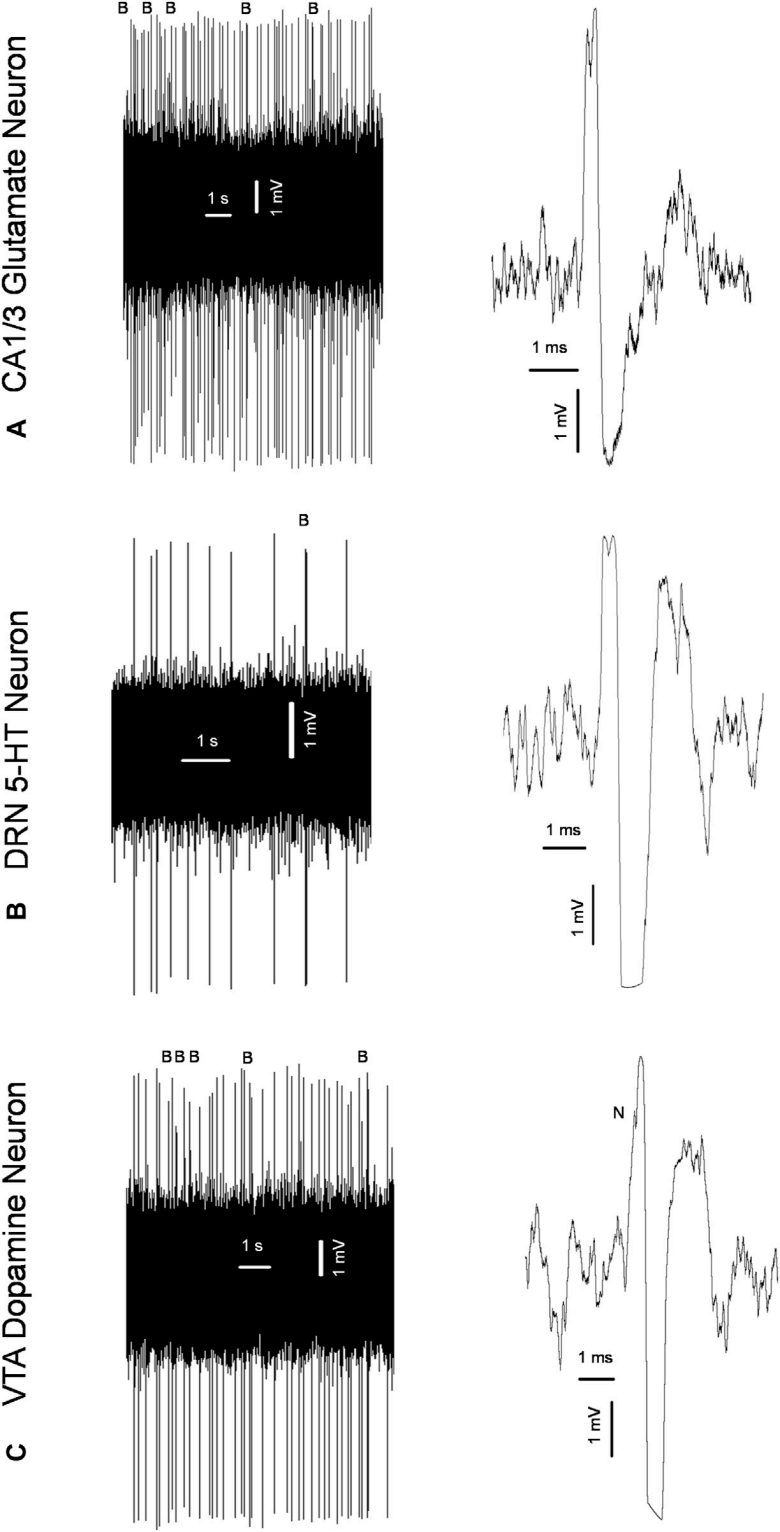
Original representative recordings from a glutamate neuron of the cornu ammonis-1/3 (CA1/3); **(A)** serotonin (5-HT) neuron of the dorsal raphe nucleus (DRN); **(B)** and dopamine neuron of the ventral tegmental area [VTA; **(C)**] from the present study, showing characteristic firing pattern (left) and waveform of the action potential (right) used for the identification of these neurons; B: bursts; N: “notch” during the rising phase of the action potential characteristic for dopamine neurons.

*In vivo* electrophysiological experiments described previously (Dremencov et al., 2022; Grinchii et al., 2022; Dremencov et al., 2023; Grinchii et al., 2023) were performed in male and female adult offspring of the SNC80- and vehicle-treated dams 24 h following behavioral testing. Urethane (1.25 g/kg, intraperitoneally: i. p.) or chloral hydrate (0.4 g/kg, i. p) anesthesia was used in experiments aiming to assess the excitability of hippocampal glutamate and brainstem monoamine neurons, respectively. The rats were mounted in the stereotaxic frame (David Kopf Instruments, Tujunga, CA), their scalp was opened, and a 3 mm hole was drilled in the skull for insertion of electrodes. Glass electrodes pulled with a DMZ-Universal Puller (Zeitz-Instruments GmbH, Martinsried, Germany) to a fine tip of ∼1 µM and filled with 2M sodium chloride (NaCl) The impedance of the electrodes was 4–6 MΩ. The electrodes were lowered through the Cornu Ammonis 1/3 (CA1/3) area of the hippocampus (3.9–4.2 mm posterior to bregma, 2.2–2.8 mm lateral to the midline, and 1.9–3.5 mm ventral to brain surface), DRN (7.8–8.3 mm posterior to bregma and 4.5–7.0 mm ventral to brain surface), or VTA (4.5–5.5 mm posterior to bregma, 0.6–0.8 mm lateral to the midline, and 7.0–8.5 mm ventral to the brain surface; Paxinos and Watson, 2014), using the hydraulic micro-positioner (David Kopf Instruments, Tujunga, CA). The action potentials generated by the neurons were recorded using the AD Instruments Extracellular Recording System (Dunedin, New Zealand). Glutamate neurons of the CA1/3, 5-HT neurons of the DRN, and dopamine neurons of the VTA were identified according to the waveform of their action potentials and the pattern of their generation (Figure 2), as explained in our previous works (Dremencov et al., 2022; Grinchii et al., 2022; Dremencov et al., 2023; Grinchii et al., 2023). During the experiment, the rats’ body temperature was maintained at 37°C with a heating pad (Gaymor Instruments, Orchard Park, NY, United States).

### 2.8 Data analysis

Action potentials (spikes) were detected using the spike sorting algorithm, with the version 6.02 of Spike2 software (Cambridge Electronic Design, Cambridge, United Kingdom). The neuronal firing rate and burst activity characteristics were calculated using the burstiDAtor software (www.github.com/nno/burstidator). The onset of the burst mode of firing and its termination were detected as previously described (Hajós et al., 2007; Csatlosova et al., 2021; Dremencov et al., 2022; Grinchii et al., 2022). Statistical assessments were performed using SigmaPlot 12.5 software (Systat Software Inc., Chicago, IL, United States). Two-way analysis of variance (ANOVA), for the factors sex (male versus female) and treatment (prenatal vehicle versus prenatal SNC80), followed by Bonferroni *post hoc* test, was used to assess the effects of the maternal SNC80 treatment and sex of the offspring on the excitability characteristics of the neurons and scores obtained in behavioural tests. Since behavior is heavily dependent on the perinatal environment, we performed the analysis by pooling offspring data from each litter and analyzing the average of each litter, to prevent confounding litter effects. For comparison, the results of statistical analyses using the values of individual animals are shown in Supplementary Material. In accordance with Wei and colleagues’ study (Wei et al., 2012), *post hoc* analysis was performed only when the ANOVA main effect of the corresponding factor or analysis or interaction between the factors were significant. Repeated measure ANOVA was used to evaluate the habituation process. The probability of *p* ≤ 0.05 was considered significant.

## 3 Results

**FIGURE 3:**
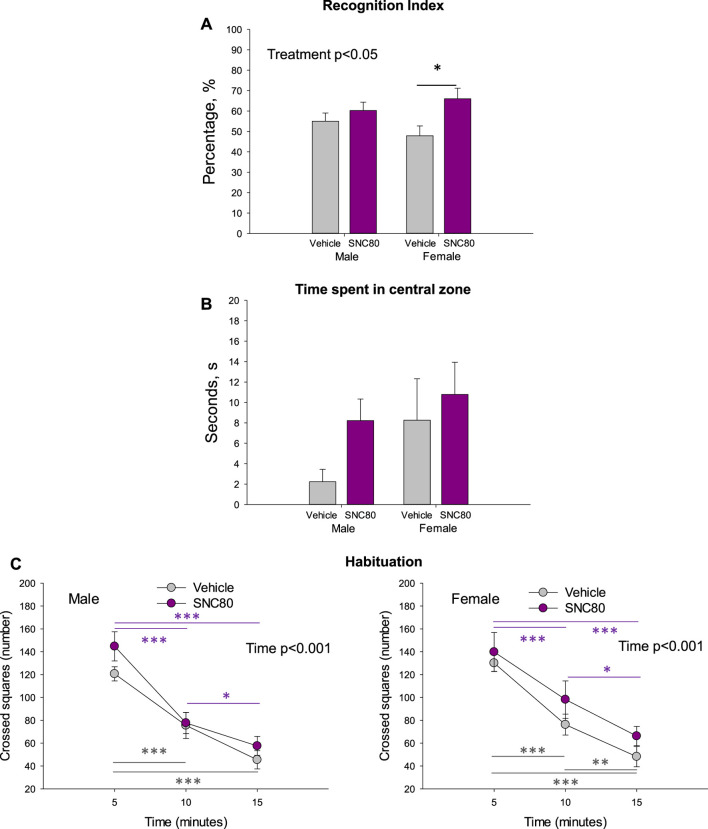
Novel object recognition index **(A)**, time spend in central zone of the open field **(B)**, and habituation behavior of the male and female offspring of the vehicle- or delta opioid agonist SNC80-treated dams **(C)**; *p ≤ 0.05, **p ≤ 0.01, and ***p ≤ 0.001, Bonferroni *post hoc* test. Were used as individual data points for statistical analysis. Are shown in the Supplementary Figure S1A.

The recognition index calculated in the offspring of SNC80-treated dams was higher than that calculated in the offspring of vehicle-treated controls in females but not males. Two-way ANOVA revealed a significant main effect of Treatment (F_1,42_ = 6.19, *p* = 0.02; data pooled per litters/dams), but no effect of sex and no sex × treatment interaction (Figure 3A). Bonferroni *post hoc* test revealed significant difference between prenatally SNC80- and vehicle-treated females (*p* = 0.03). Data based on individual animals used as the values for statistical analysis are shown in the Supplementary Figure S1A.

Prenatal treatment with SNC80 failed to significantly modify anxiety behaviour as measured in the OF test (time spent in the central zone). When data was pooled per litters/dams, no effects of sex, treatment, or sex × treatment interaction were observed (Figure 3B). When the individual animals were used as data points for statistical analysis, two-way ANOVA showed a marginally significant main effect of sex (F_1,36_ = 3.991, *p* = 0.05), but still no effect of treatment and sex × treatment interaction (Supplementary Figure S1B). Female offspring regardless of treatment spent a longer time in the central zone of the OF than males.

Prentatal exposure to maternal SNC80 treatment failed to modify the rapidity of habituation (k-values) in the open field in the offspring (3C). By evaluating the number of squares crossed in the consecutive three 5-min time periods in the open field, two-way ANOVA for repeated measures (data polled per litters/dams) revealed a significant main effect of time (males: F_1,44_ = 83.13, *p* < 0.001; females: F_1,29_ = 88.12, *p* < 0.001) but no effect of treatment and or sex × treatment interaction. In all groups, the number of squares crossed decreased in time. Data based on individual animals used as the values for statistical analysis are shown in the Supplementary Figure S1C.

**FIGURE 4:**
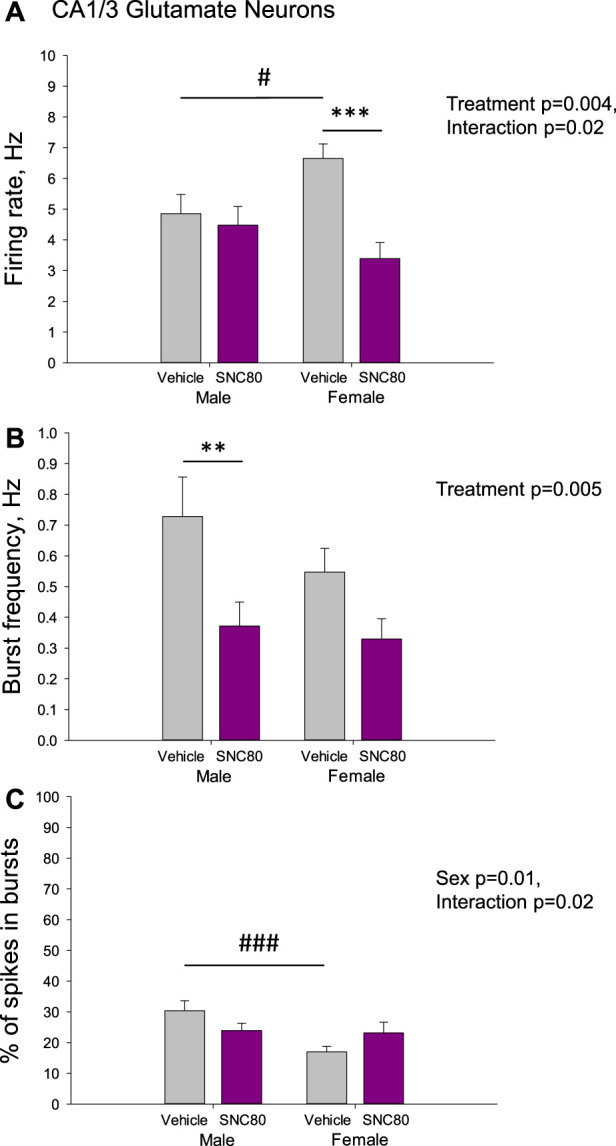
Firing rate **(A)** and characteristics of the burst firing **(B, C)** of glutamate neurons of the cornu ammonis-1/3 (CA1/3) in the male and female offspring of the vehicle- or delta opioid agonist SNC80-treated dams; **p ≤ 0.01 and ***p ≤ 0.001 in comparison with the same-sex offspring of the vehicle-treated dams; ^#^ *p* ≤ 0.05 and ^###^ *p* ≤ 0.001 in comparison with the males of the same treatment group, Bonferroni *post hoc* test.

Figure 4 illustrates the excitability of glutamate neurons of the CA1/3 in male and female rats prenatally exposed to the vehicle or SNC80. Female (*n* = 36 neurons from five rats), but not male (*n* = 93 neurons from eight rats) offspring of SNC80-treated dams exhibited lower mean basal firing rate of CA1/3 glutamate neurons compared to that in offspring of the vehicle-treated controls (*n* = 64 neurons from six male and 100 neurons from 10 female rats; A). Two-way ANOVA showed significant effect of the treatment (F_1,292_ = 8.23, *p* = 0.004), significant sex × treatment interaction (F_1,292_ = 5.18, *p* = 0.02), but no effect of sex. Bonferroni *post hoc* test revealed significant difference between prenatally SNC80- and vehicle-treated females (*p* < 0.001) and between prenatally vehicle-treated males and females (*p* = 0.03) with higher values in female rats.

The frequency of the bursts was lower in offspring of SNC80-treated dams compared to that in offspring of vehicle-treated controls, but in male rats only (B). Two-way ANOVA showed significant main effect treatment (F_1,249_ = 8.17, *p* = 0.005), but no effect of sex or sex × treatment interaction. Bonferroni *post hoc* test revealed significant difference between prenatally SNC80- and vehicle-treated males (*p* = 0.007).

With respect to the percentage of spikes occurring in the bursts, this parameter was lower in female than in male offspring of the control dams and this sex difference disappeared in offspring of the SNC80-treated dams (C). Two-way ANOVA showed significant effect of sex (F_1,249_ = 6.59, *p* = 0.01) and sex × treatment interaction (F_1,249_ = 5.23, *p* = 0.02), but no effects of treatment. Bonferroni *post hoc* test revealed significant difference between prenatally vehicle-treated males and females (*p* < 0.001).

**FIGURE 5:**
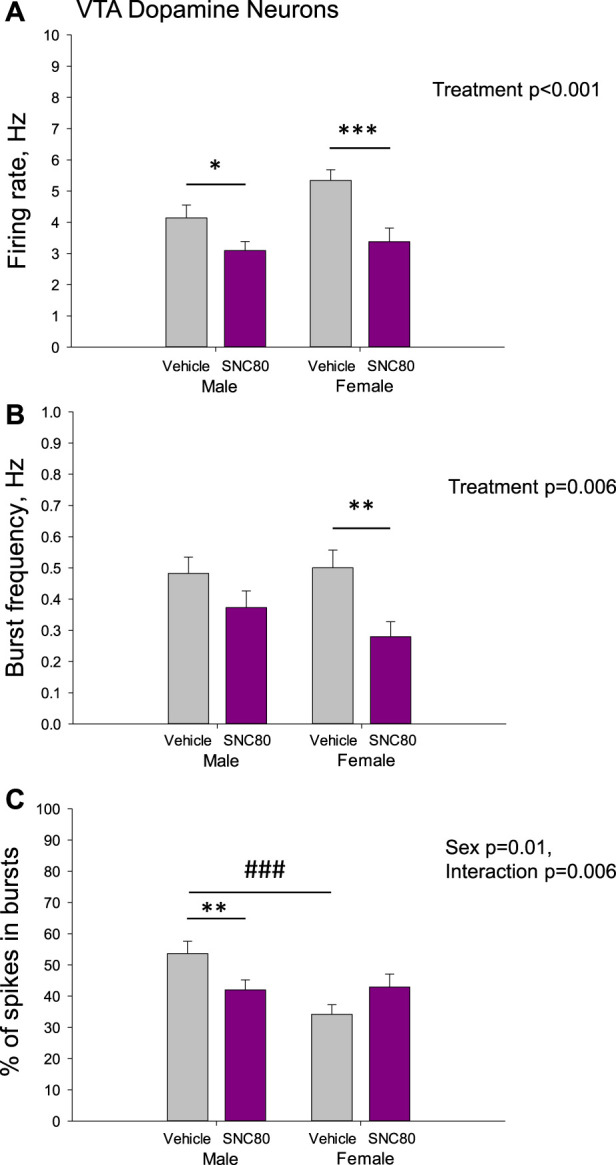
Firing rate **(A)** and characteristics of the burst firing **(B, C)** of dopamine neurons of the ventral tegmental area (VTA) in the male and female offspring of the vehicle- or delta opioid agonist SNC80-treated dams; *p ≤ 0.05, **p ≤ 0.01, and ***p ≤ 0.001 in comparison with the same-sex offspring of the vehicle-treated dams; ^###^ *p* ≤ 0.001 in comparison with the males of the same treatment group, Bonferroni *post hoc* test.

Figure 5 illustrates the excitability of dopamine neurons of the VTA in male and female rats prenatally exposed to vehicle or SNC80. Male (*n* = 77 neurons from six rats) and female (*n* = 40 neurons from five rats) offspring of SNC80-treated dams exhibited lower mean basal firing rate of VTA dopamine neurons, comparing to that in offspring of the vehicle-treated controls (*n* = 52 neurons from five male and 93 neurons from female 12 rats; A). Two-way ANOVA showed significant main effect of treatment (F_1,261_ = 15.58, *p* < 0.001), but no effect of sex and no sex × treatment interaction. Bonferroni *post hoc* test revealed significant difference between prenatally SNC80- and vehicle-treated males (*p* = 0.05) and between prenatally SNC80- and vehicle-treated females (*p* < 0.001).

With respect to the burst frequency, it was lower in female, but not in male offspring of SNC80-treated dams, comparing to that in offspring of the vehicle-treated controls (B). Two-way ANOVA showed significant main effect of the treatment (F_1,261_ = 7.61, *p* = 0.006), but no effect of sex and no sex × treatment interaction. Bonferroni *post hoc* test revealed significant difference between prenatally SNC80- and vehicle-treated females (*p* = 0.01).

The percentage of spikes occurring in the burst was lower in the male, but not female offspring of SNC80-treated dams, comparing to that in offspring of the vehicle-treated controls(C). Two-way ANOVA showed significant effect of sex (F_1,261_ = 6.29, *p* = 0.01) and sex × treatment interaction (F_1,261_ = 7.58, *p* = 0.006), but no effect of treatment. Bonferroni *post hoc* test revealed significant difference between prenatally SNC80- and vehicle-treated males (*p* = 0.01), as well as between prenatally vehicle-treated males and females (*p* < 0.001) with higher values in males.

**FIGURE 6:**
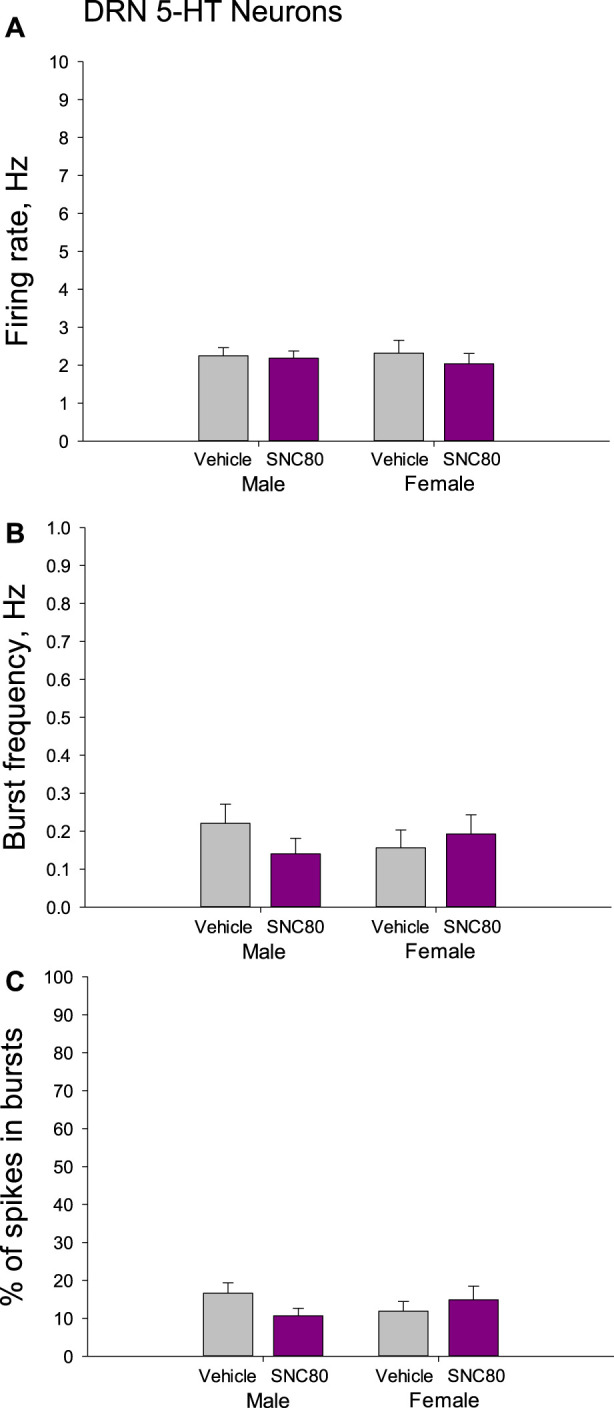
Firing rate **(A)** and characteristics of the burst firing **(B, C)** of serotonin (5-HT) neurons of the dorsal raphe nucleus (DRN) in the male and female offspring of the vehicle- or delta opioid agonist SNC80-treated dams.

There were no differences in the mean basal firing rate of 5-HT neurons of the DRN between males and females or between the offspring of SNC80 (n = 71 neurons from 7 to 50 neurons from six rats, respectively)- and vehicle (n = 59 neurons from 9 to 36 neurons from four rats, respectively)-treated dams. The characteristics of the burst firing of 5-HT neurons, such as frequency of the bursts and percent of spikes occurring in the bursts, were not different between the sexes and between the treatment groups as well (Figure 6).

## 4 Discussion

The present results demonstrate that the treatment with a delta-opioid agonist during gestation does not induce negative effects on anxiety, cognitive performance or habituation processes in coping with new environment in the offspring. Moreover, female rats prenatally exposed to maternal treatment with a delta-opioid agonist showed a better cognitive performance in a NOR test. Consistently, the decrease in firing of glutamate and dopamine neurons as well as the burst frequency of dopamine neurons observed in female offspring prenatally exposed to maternal treatment with a delta-opioid agonist was less pronounced or absent in male ones.

Offspring of dams treated with the delta-opioid agonist during gravidity did not show any negative changes in the behavioral tests used in present experiments. In existing studies on prenatal opioid exposure, the authors examined mainly the effects of heroin, methadone or buprenorphine showing many negative neurodevelopmental alterations in the offspring (Conradt et al., 2019; Lee et al., 2023). The treatment with the delta-opioid agonist SNC80 in the present study failed to negatively modify the offspring behavior. To our best knowledge, there are no other studies exploring the consequences of maternal treatment with selective delta-opioid agonists on offspring behavior.

Interestingly, female offspring of SNC80-treated dams showed even a better cognitive performance compared to that in female offspring of vehicle-treated dams. Though the data on effects of prenatal exposure are missing, the present data are consistent with positive effects of delta-opioid agonists on cognitive performance in adults. As mentioned in the introduction, delta-opioid agonists can positively modulate several processes of brain plasticity, such as cell proliferation or BDNF expression (Persson et al., 2003a; Torregrossa et al., 2006). It was also shown that the activation of delta-opioid receptors in adult rats experienced hypoxia improve spatial cognition, and hippocampal neurogenesis and synaptic transmission (Wang et al., 2016; Zhang et al., 2021). Treatment of adult rats with delta-opioid agonists appears to induce mild anxiolytic effects (Saitoh et al., 2004; Dremencov et al., 2023). However, as shown in the present study, such effects were not transferred to the offspring. Electrophysiological measurements in glutamate neurons have shown that the treatment with SNC80, a delta-opioid agonist, during gestation resulted in decreased firing rate in female and suppressed burst activity in male offspring. A clear inhibitory effect of prenatal exposure to maternal SNC80 treatment on both firing rate and burst frequency of dopamine neurons were observed in female offspring. In male offspring of dams treated with SNC80, the firing rate and the percentage of spikes in bursts were lower compared to corresponding values in control offspring. Chronic treatment with the same delta-opioid agonist during adulthood in our previous study (Dremencov et al., 2023) led to an opposite effect, namely, increased firing rate of glutamate and dopamine neurons in male rats. In the mentioned study, the SNC80 effects in female rats were however not investigated. The present data cannot be discussed based on previous studies on prenatal selective delta-opioid agonists, as such studies could not be found in the literature available.

It is known that the burst firing of glutamate (Wozny et al., 2008) and dopamine (Cooper, 2002) neurons enhances the nerve terminal neurotransmitter release, in comparison with the same amount of action potentials fired in a single-spike mode. Suppression of the burst firing of glutamate and dopamine neurons in the offspring of dams treated with SNC80-treated dams may thus result in a further decrease in corresponding neurotransmission.

The activation of maternal delta-opioid receptors during gestation had no effect on characteristics of the firing activity of 5-HT neurons of the DRN in their offspring. Apparently, serotoninergic neurotransmission is not affected by prenatal exposure to delta-opioid agonist treatment.

Improved NOR performance in female offspring of delta opioid agonist-treated rats might be associated with decreased firing rate of hippocampal glutamate and mesolimbic dopamine neurons. The present findings are supported by the results of a previous study showing a link between elevated hippocampal glutamate neurotransmission and impaired NOR performance (Gómez-Galán et al., 2013). Furthermore, a recent study describes that enhanced cognitive performance induced by exposure of rodents to multiple NOR sessions was accompanied by decreased activity of VTA dopamine neurons, measured indirectly by c-Fos immunohistochemistry (Fleury et al., 2023).

With respect to the sex differences, the time spent in the central zone measured in the open field test (anxiety behavior) was marginally longer in female than in male offspring. That agrees with the known high exploratory activity in the open field in females (Dubovický et al., 1999).

Interestingly, the firing rate of both glutamate and dopamine neurons was higher and the percentage of spikes occurring in burst was lower in males compared to females. Observed sex differences in the firing activity of dopamine neurons are in agreement with a similar tendency found in our previous experiments (Csatlosova et al., 2021). A recent study from a different laboratory (Zhu and Grace, 2023), however, reported no differences in the firing activity of hippocampal glutamate and VTA dopamine neurons between males and females under basal conditions. The mentioned authors used however a different strain of rats. In agreement with our previous results (Csatlosova et al., 2021) there were no sex differences in the firing activity of 5-HT neurons. The characteristics of the firing activity of hippocampal glutamate and brainstem 5-HT and dopamine neurons in offspring of the control dams were similar to those observed in control animals in our previous studies without any behavioral testing (Grinchii et al., 2022; Grinchii et al., 2023). Even though in the present study behavioral tests were performed on the same animals before electrophysiological recordings, they are unlikely to affect the firing activity of the neurons.

Maternal treatment with delta-opioid agonists can alter the excitability of the hippocampal glutamate and VTA dopamine neurons via several possible mechanisms. First, these drugs are likely to pass the placenta and to affect the embryonal brain directly. Their ability to alter the excitability of hippocampal glutamate and VTA dopamine neurons was shown in our previous studies (Dremencov et al., 2023). Second, activation of delta-opioid receptors may affect the expression of certain maternal neurotrophic factors, such as BDNF (Torregrossa et al., 2006). Maternal neurotrophins are in turn suggested to interact with the embryonal ones and to affect developmental and functioning of the embryonal brain (Singh et al., 2022). Finally, delta agonists can affect the maternal care behavior that may influence offspring behavior and neurophysiology (Gemmel et al., 2018). Further studies should be performed to assess the mechanism(s) interconnecting maternal treatment with delta-opioid agonists and the activity of the hippocampal glutamate and VTA dopamine neurons in offspring.

There are a few limitations of the present study. As the results were evaluated using *post hoc* analyses even when the interaction between the main factors was not statistically significant, the conclusions were formulated with caution.

In conclusion, the original findings of the present study suggest that the maternal treatment with delta-opioid agonists during the gestation may have pro-cognitive effect in female offspring. Observed pro-cognitive effect induced might be mediated via mechanism(s) involving the firing activity of hippocampal glutamate and mesolimbic dopamine neurons.

## Data Availability

The raw data supporting the conclusion of this article will be made available by the authors, without undue reservation.
