# Supplementary material for: Maternal treatment with a selective delta-opioid receptor agonist during gestation has a sex-specific pro-cognitive action in offspring: mechanisms involved
Source: Front Pharmacol. 2024 Apr 16;15:1357575. doi: 10.3389/fphar.2024.1357575 (PMC11059060; doi:10.3389/fphar.2024.1357575)
Supplement: Supplementary file 1 [file DataSheet1.PDF]

Figure S1: Novel object recognition index (A), time spent in central zone of the open field (B), and habituation behavior (C) of the male and female offspring of the vehicle- or delta opioid agonist SNC80-treated dams; *$p \leq 0.05$, **$p \leq 0.01$, and ***$p \leq 0.001$, Bonferroni post-hoc test. Rats were used as individual data points for statistical analysis. Two-way ANOVA revealed a significant main effect of treatment ($F_{1,215}$=6.36, p=0.01) on the time spent in the central zone, but no effect of sex and no sex × treatment interaction. With respect to the time spent in the central zone, two-way ANOVA showed a marginally significant main effect of sex ($F_{1,36}$=3.991, p=0.05), but no effect of treatment or sex × treatment interaction. With respect to the habituation, repeated measure ANOVA (Figure 3C) revealed a significant main effect of time but no effect of treatment and sex.

Figure S1

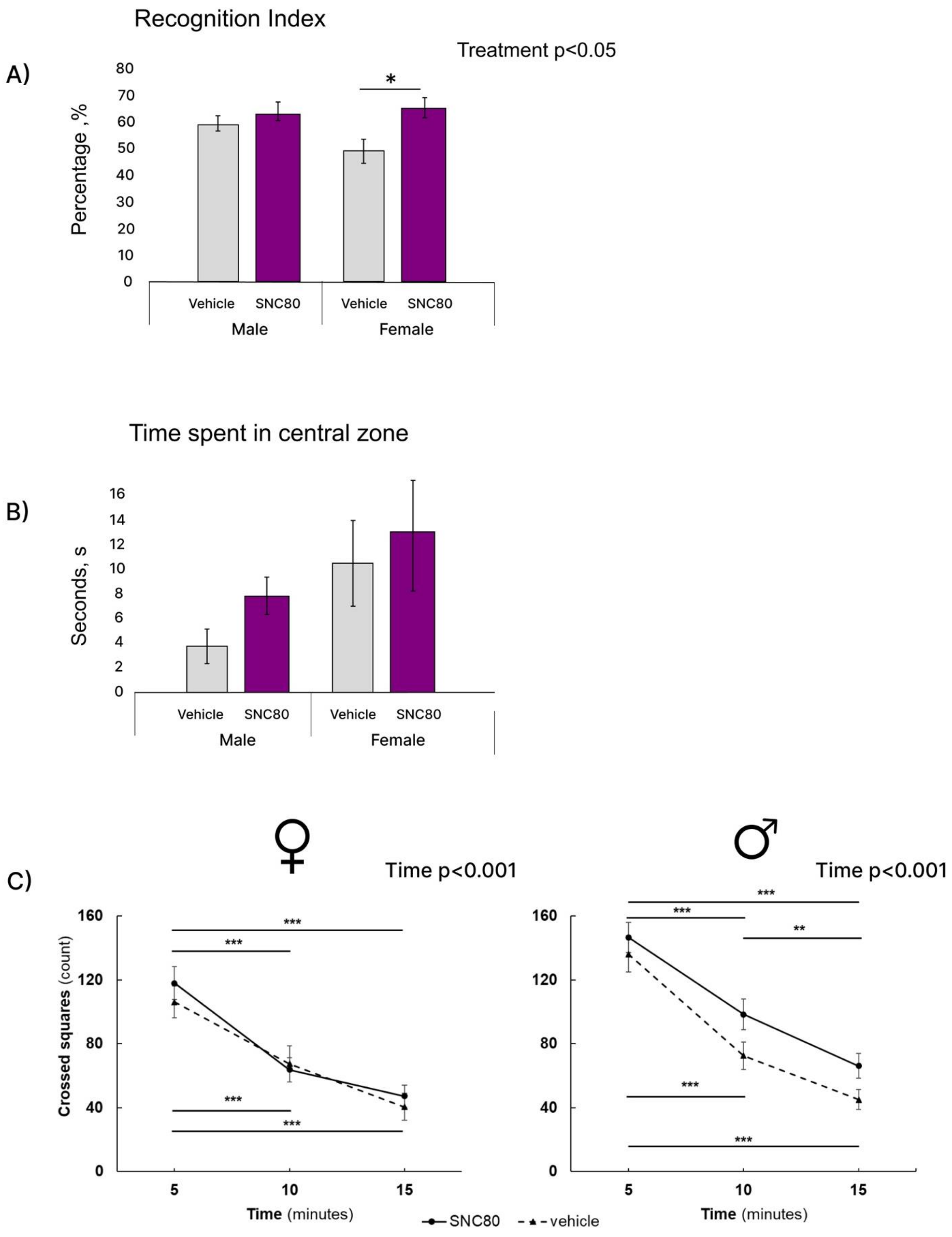
Recognition Index
Treatment p<0.05
A)
Percentage ,%
80
70
60
50
40
30
20
10
0
*
Vehicle
SNC80
Vehicle
SNC80
Male
Female
Time spent in central zone
B)
Seconds, s
16
14
12
10
8
6
4
2
0
Vehicle
SNC80
Vehicle
SNC80
Male
Female
♀
♂
C)
Time p<0.001
Time p<0.001
Crossed squares (count)
160
120
80
40
0
***
**
5
10
15
Time (minutes)
SNC80
vehicle
